# A Case Report of Dermatofibrosarcoma Protuberans and Refractory Hypomagnesemia: Unveiling a Paraneoplastic Syndrome

**DOI:** 10.7759/cureus.55301

**Published:** 2024-03-01

**Authors:** Pranav Chaudhari, Rucha Sawant, Nikhil Reddy, Sunil Kumar, Sourya Acharya

**Affiliations:** 1 Department of Medicine, Jawaharlal Nehru Medical College, Datta Meghe Institute of Higher Education and Research, Wardha, IND

**Keywords:** radiotherapy, magnesium, magnesium deficiency, tumor, dermatofibrosarcoma protuberan, paraneoplastic syndromes

## Abstract

Dermatofibrosarcoma protuberans (DFSP) is a rare, locally aggressive soft tissue sarcoma of the skin. DFSP typically presents as an asymptomatic, firm plaque in its earliest stage, gradually enlarging over months to years. This case report delineates a singular instance involving a 66-year-old female patient previously diagnosed with and treated for DFSP. The individual presented with a recurrent history of hospital admissions, manifesting symptoms of loose stools, generalised weakness, and diminished appetite. Investigations revealed persistent hypomagnesemia. The patient was treated with magnesium correction. Eventually, all complaints were resolved, and the patient was discharged satisfactorily. This case report aims to elucidate an exceptional correlation between DFSP and hypomagnesemia, characterised as its paraneoplastic syndrome (PNS). This study intends to comprehensively outline the clinical manifestations, diagnostic approaches, and therapeutic interventions pertaining to this distinct association.

## Introduction

Over a century ago, doctors observed unsettling symptoms in some cancer patients, seemingly uncoupled from tumor growth itself [1]. These enigmatic occurrences, christened "paraneoplastic syndromes" in the 1940s, remained in mystery for decades [1]. Today, the spotlight shines on two critical actors in this puzzling drama: rogue hormones secreted by tumors and mistaken identity by the immune system, attacking healthy tissues. Recent medical advancements have shed light on the intricate workings of these syndromes, paving the way for improved diagnosis and treatment.

Remarkably, some paraneoplastic syndromes act as harbingers, presenting before a cancer diagnosis. Recognizing them promptly can unearth hidden tumors at an early, highly treatable stage. This is particularly true for neurological paraneoplastic disorders. While distinguishing them from similar non-cancerous conditions has long been challenging, an arsenal of blood tests and imaging techniques now aids in this crucial task. Estimates suggest that up to 8% of cancer patients encounter these enigmatic syndromes [1]. This number will likely rise as cancer patients live longer, and diagnostic tools become more refined. However, the rarity of individual syndromes poses a challenge - large-scale clinical trials for specific management strategies are scarce. Nonetheless, paraneoplastic syndromes often correspond to subcategories of illnesses that also happen independently of a cancer relationship. This review mixes clinical data from case series of particular paraneoplastic illnesses and broader studies of clinically comparable, nonparaneoplastic ailments to offer an overview of diagnosing and managing the most often seen paraneoplastic syndromes.

Darier initially reported dermatofibrosarcoma protuberans (DFSP), an unusual cutaneous fibrohistiocytic tumor, in 1924 [2], and Hoffman first used the term "DFSP" in 1925 [3]. One rare instance of a locally invasive dermatological soft tissue sarcoma is DFSP. Eight to nine out of 10 cases of DFSPs are low grade [4].

Since the turn of the century, cases have been reported to have inadequate amounts of magnesium [5]. Even yet, magnesium has earned the moniker "forgotten cation" as Mg2+ availability is seldom assessed and tracked in individuals, despite the mineral's widely acknowledged significance. Mg2+ is a critical component in the multistage, multifactor-related process of malignancy [6].

## Case presentation

A 66-year-old female, who was a known and treated case of DFSP, came to the casualty with complaints of recurrent bouts of loose stools with six to seven episodes of non-sticky, non-blood stained, watery stools and had been hospitalized thrice within a span of two months for the same. Examination revealed that the patient had a blood pressure of 90/60 mmHg and a pulse rate of 108/min. Signs of dehydration were visible, such as sunken eyes and poor skin turgor. Cardiovascular and abdominal examinations were normal. Local examination at the site of malignancy was normal. Laboratory investigation revealed routine levels of complete blood count, liver and renal function test, electrolytes (sodium of 140 mg/dL, potassium of 4.0 mg/dL, calcium of 9.0 mg/dL, phosphorus of 3.4 mg/dL, chloride of 104 mg/dL), with the exception low magnesium (i.e., 1.2 mg/dL; Table 1).

**Table 1:** Laboratory investigations.

PARAMETERS	VALUES	NORMAL RANGE
Haemoglobin (gm/dL)	9.3	11-15
Total White Blood Cell Count (per cu.mm)	5000	4000-10,000
Total Red Cell Count (million per cu.mm)	3.16	4.2-5.4
Platelets (per cu.mm)	243,000	140,000-440,000
Haematocrit (%)	29.4	36-46
Urea (gm/dL)	22	10-45
Creatinine (mg/dL)	0.7	0.2-1.2
Sodium (meq/dL)	140	135-148
Potassium (meq/dL)	4.3	3.5-5.3
Magnesium (mg/dL)	1.2	1.6-2.3
Calcium (mg/dL)	8.2	8.4-10.2
Phosphorus (mg/dL)	3.3	2.5-4.5
Uric acid (mg/dL)	5.2	2.5-6.5
RBS (mg/dL)	91	<140
Serum Glutamyl-Oxaloacetate Transaminase (U/L)	24	11.8-64.8
Serum Glutamyl- Pyruvate Transaminase (U/L)	14	8.5-49.5
Albumin (gm/dL)	3.4	3.2-5.1
Total Bilirubin (gm/dL)	0.5	0-0.8
International Normalised Ratio (INR)	1.05	1.3-1.5

**Figure 1:**
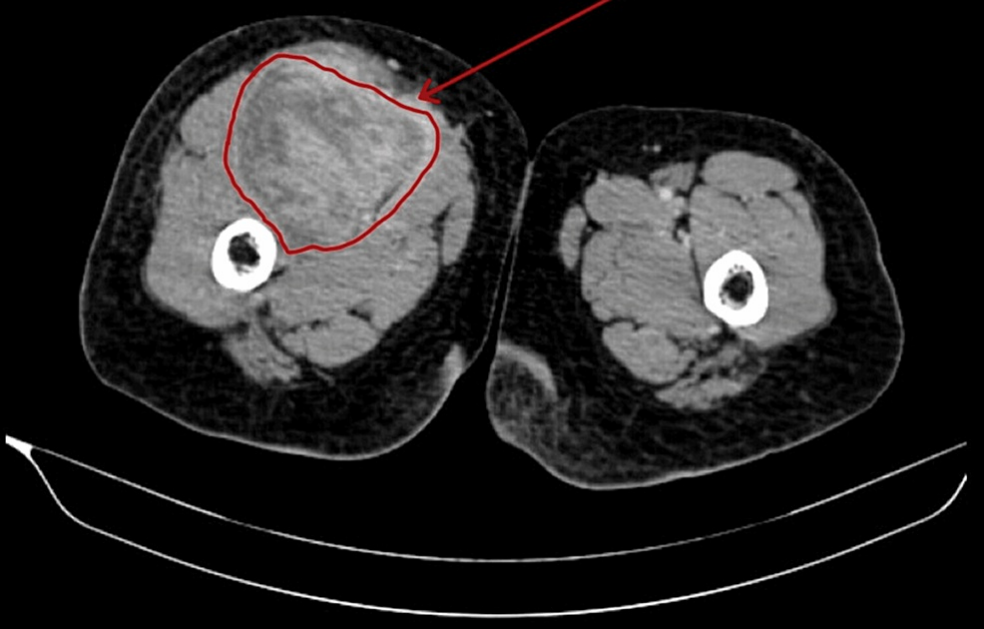
Contrast-enhanced CT thigh venous phase showing a large, well-defined lobulated lesion of size 12.5 x 8 x 8 cm in the intermuscular plane of the anterior aspect of the right upper thigh, along with compression of adjacent muscles.

**Figure 2:**
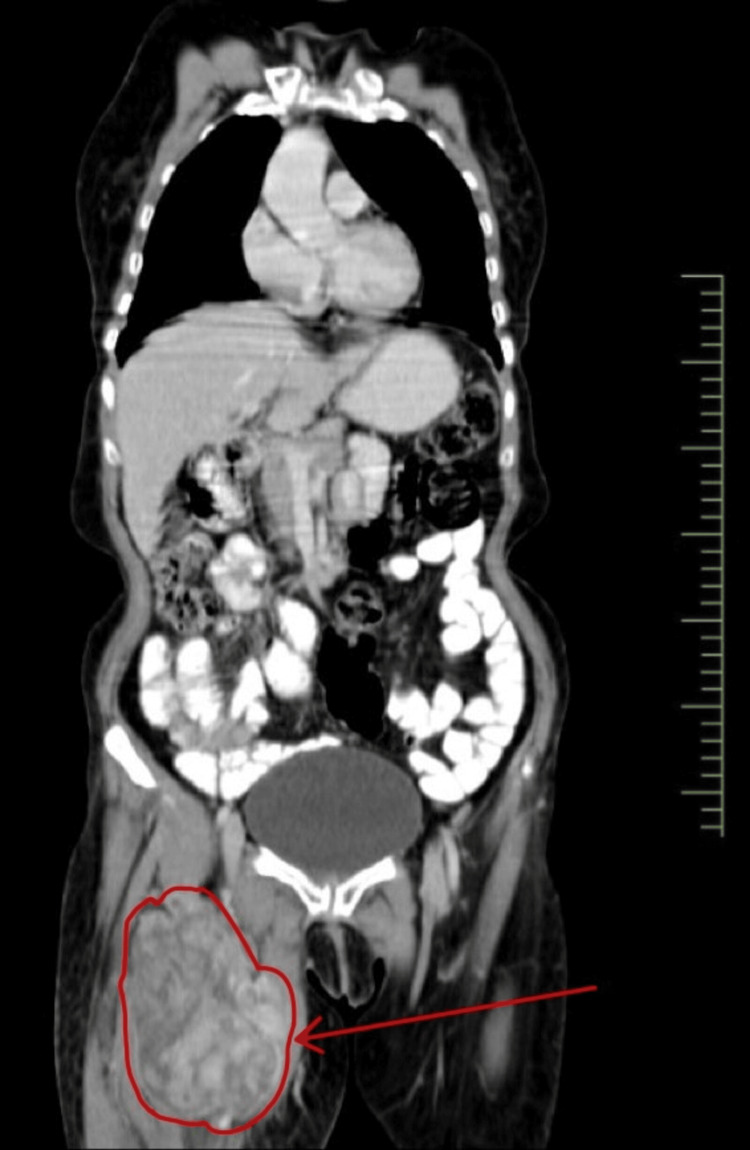
Contrast-enhanced CT showing an ill-defined heterogeneously enhancing lesion measuring 13 x 8.5 cm in the right proximal thigh.

**Figure 3:**
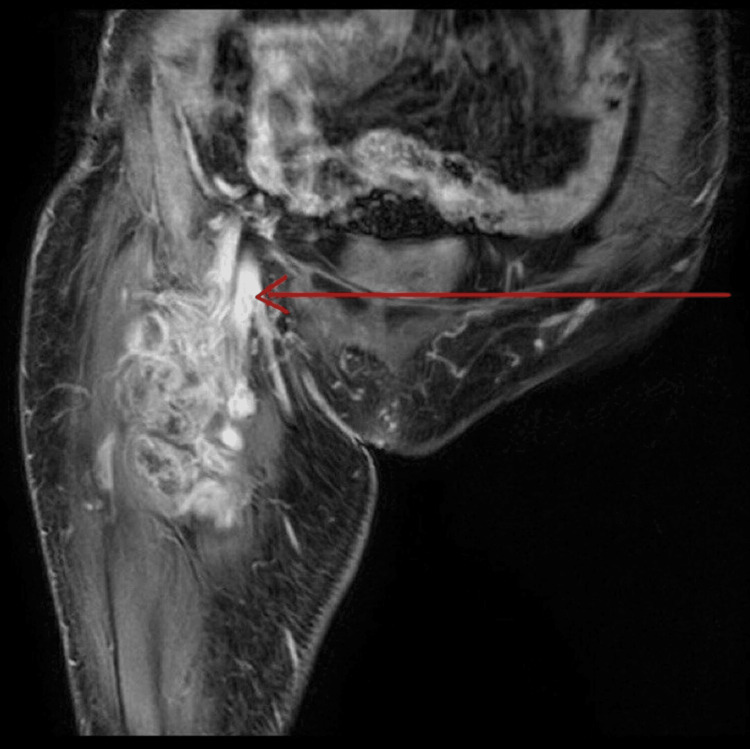
T1-weighted coronal section post-contrast image showing evidence of an altered signal intensity lesion in the intermuscular plane of the right thigh involving the vastus intermedius, vastus medialis, and distal iliopsoas muscles showing moderate post-contrast enhancement. The lesion is encasing deep profunda femoral vessels and femoral nerve.

**Figure 4:**
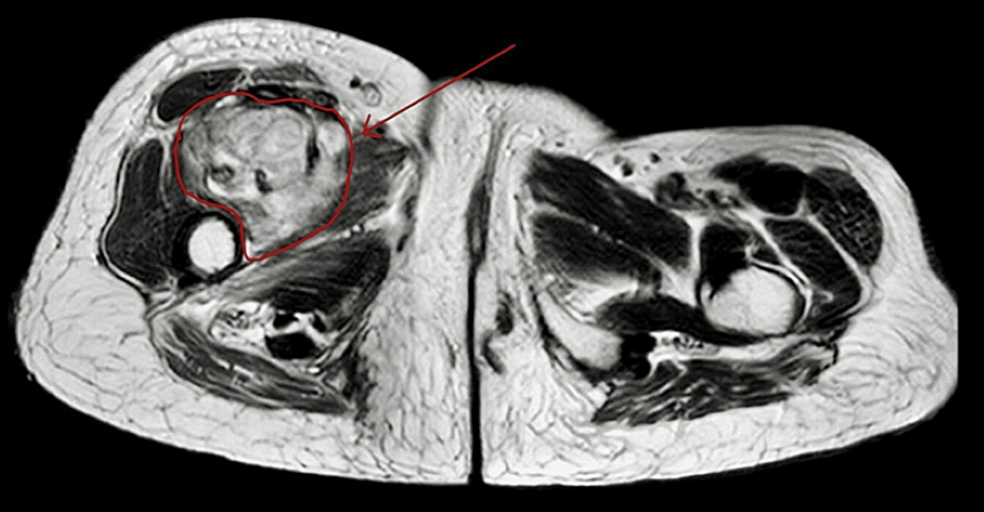
T2-weighted image showing a heterogeneously hyperintense lesion in the intramuscular plane of the right thigh (red arrow).

**Figure 5:**
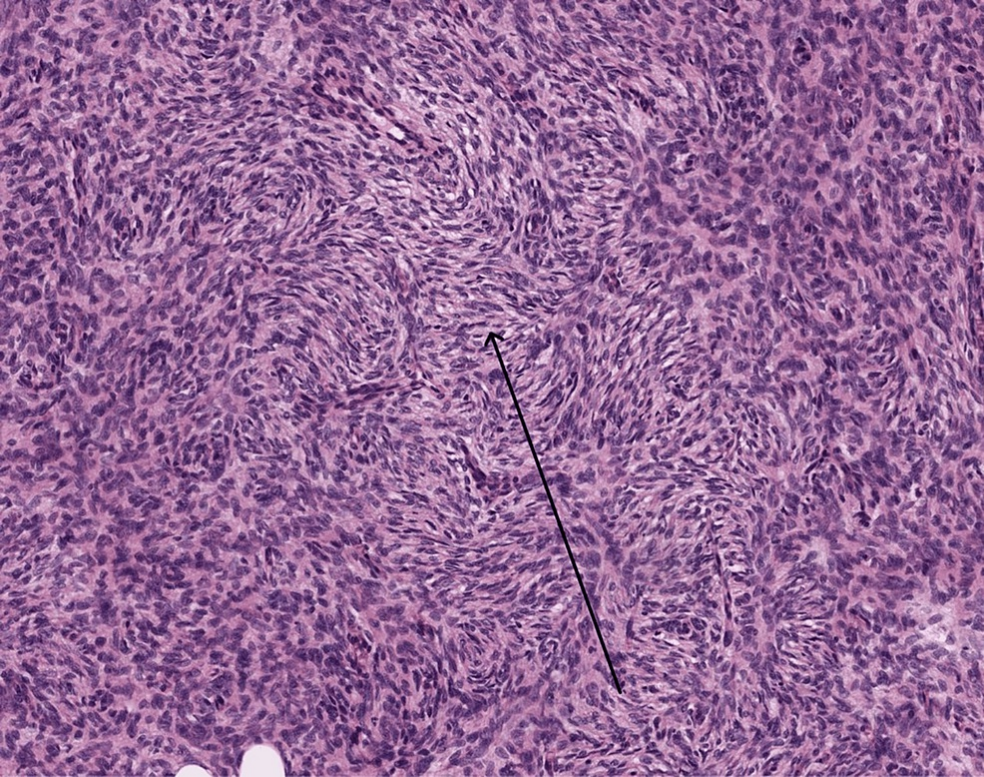
High magnification demonstrating spindle cells with a storiform pattern.

The patient was first diagnosed with DFSP in November 2022 when she noticed a lump over her right thigh and visited the hospital for the same. Contrast-enhanced CT (Figures 1-2) and contrast-enhanced MRI of the right thigh were made, which suggested a large, well-defined lobulated lesion of 12.5 x 8.2 x 8 cm in the anterior aspect of the right upper thigh, encasing the femoral vessels and nerves (Figures 3-4). A biopsy was taken, and a report of histopathology (Figure 5) and immunohistochemistry indicated vimentin-positive and S-100 negative. Taking into account her clinical presentation, she was diagnosed with non-metastatic mesenchymal tumor DFSP with encasement of femoral vessels (intermediate malignant potential). The radio oncologist started her on neoadjuvant radiotherapy with a dose of 50.4 Gy in 28 fractions using the three-dimensional (3D) conformal radiation therapy (CRT) technique. She completed 28 fractions after a month of starting radiotherapy, following which she was advised surgery, for which she refused, citing financial reasons.

**Figure 6:**
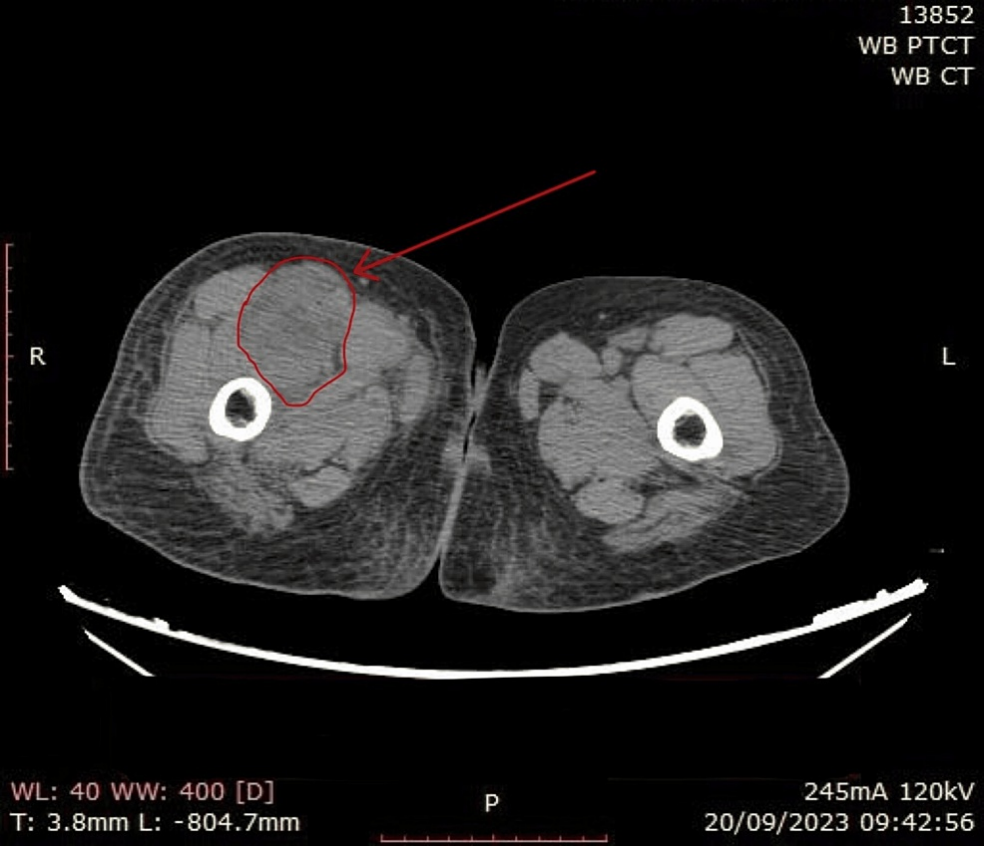
Whole-body PET-CT scan CT image showing a soft tissue mass in the right anterior compartment of the thigh.

After six months of stopping radiotherapy, the patient developed her first bouts of loose stools, for which she was hospitalized. Reports suggested low magnesium of 1.2 mg/dL (normal lab values: 1.6-2.3 mg/dL); hence, she was treated with magnesium correction after ruling out renal causes of hypomagnesemia. The magnesium was given as an infusion of 8 gm of magnesium sulfate over 12 hours. The patient was discharged with a normal magnesium value and no complaints, and after 20 days, the patient came to the OPD with similar complaints. To rule out residual disease and metastasis, the patient was advised a whole-body PET-CT scan, which suggested a soft tissue mass in the right anterior compartment of the thigh measuring 5.3 x 4.5 x 8 cm with mildly increased metabolism, likely residual disease with no active disease elsewhere and no evidence of distant metastasis (Figure 6). The oncologist's opinion was taken, and conservative management was advised as the patient was not willing to have surgery or chemotherapy due to personal, financial, and psychological reasons. During her second hospitalization for loose stools, her electrolytes were tested and suggested hypomagnesemia. Her condition was treated as earlier, and she was discharged with no complaints.

Within 15 days of her discharge, she presented with similar complaints to the casualty again. The patient was treated symptomatically and started on low-dose magnesium supplementation with 185 mg of magnesium hydroxide twice daily. To rule out other causes of secretory diarrhea, an upper gastrointestinal endoscopy and colonoscopy with biopsy were done, which suggested no apparent abnormality. The patient was discharged with no complications or complaints. The patient was kept on magnesium supplementation at home, and a three-month follow-up did not show any similar episodes or hospitalizations for a similar cause. Since the patient’s complaints were resolved on magnesium treatment, it was concluded that loose stools were not the sole cause, but rather an effect of hypomagnesemia. Hence, the patient was diagnosed with hypomagnesemia as PNS of DFSP.

## Discussion

In our patient, we diagnosed hypomagnesemia as PNS of DFSP as the patient presented thrice with complaints of loose stools, which was associated with hypomagnesemia and was resolved on serum magnesium correction. We have concluded that hypomagnesemia was the cause of loose stools as PNS of DFSP rather than the effect of loose stool. In this discussion, we discuss magnesium and its function in the human body, the impact of hypomagnesemia, and its role in carcinogenesis.

Physiological processes govern the amount of magnesium in the body: gastrointestinal (GI) uptake, kidney reabsorption/excretion, and regulation from the bodily Mg2+ (bones). Under normal circumstances, the relationship between GI uptake and kidney elimination carefully regulates the stocks of Mg2+. Naturally, the kidneys eliminate more magnesium when there is an excess. When there is a deficiency, the amount of magnesium in the urine can drop to as little as 1 mEq (~12 mg). Similar to calcium, magnesium can be obtained from the bone, muscles, and internal organs when intakes are inadequate to maintain normal blood magnesium levels despite renal conservation [5].

Separating the causes of hypomagnesemia into renal and GI losses is helpful. Diarrhea is a common cause of intestinal magnesium loss in cancer patients. Healthcare providers should be mindful that extended and chronic utilization of proton pump inhibitors may lead to hypomagnesemia, primarily when used in conjunction with diuretics [7]. The differentiation between Mg2+ losses through the renal and GI tract can be facilitated by assessing either the 24-hour urinary Mg2+ level or the fractional excretion of Mg2+ in a random urine sample. Hypomagnesemia resulting from impaired renal handling is linked to abnormally high urine magnesium levels [7]. Sepsis and diabetes are independent risk factors for developing hypomagnesemia [8]. Hypomagnesemia is also associated with DM, possibly due to increased renal losses of Mg accompanying glycosuria. There is also a strong relationship between hypomagnesemia and insulin resistance, and Mg2+ supplementation is associated with decreased insulin requirements [9]. It has been reported in several cases that some neuroendocrine tumors have been implicated in causing secretory diarrhea, which in turn causes hypomagnesemia [10]. Excess serotonin increases peristalsis, reducing water absorption and electrolytes and leading to diarrhea [11]. Another example of tumor-induced diarrhea is that of a carcinoid tumor that is associated with serotonin syndrome [12,13].

Management for hypomagnesemia can be complex, particularly if the renal loss is continuing. While high plasma magnesium may decrease renal reabsorption, symptomatic patients with clear indications should still receive intravenous magnesium therapy. Up to half of the injected magnesium can be excreted in the urine, necessitating slower infusion rates and close monitoring of serum levels for adequate correction. Due to the limited bioavailability and diarrhea-inducing potential of oral magnesium salts, achieving the desired correction might be challenging. Emerging evidence suggests that sodium-glucose co-transporter type 2 (SGLT2) inhibitors, which primarily work by lowering blood sugar through increased urinary glucose excretion, may also have the unintended benefit of increasing magnesium levels in those suffering from hypomagnesemia [7]. Although SGLT2 inhibitors have emerged as a potential avenue for managing hypomagnesemia, further studies and clinical experience are required before they can be regarded as standard care. These may include a reduction in the cotransport of sodium and glucose in the proximal tubule, a potential decrease in extracellular fluid volume, which activates the renin-angiotensin-aldosterone pathway, thus enhancing renal magnesium reabsorption and possibly the expression of renal or GI magnesium channels.

## Conclusions

Through this case, we have concluded a rare correlation between hypomagnesemia and PNS of DFSP. The intriguing part was whether hypomagnesemia manifested as a PNS, the culprit behind the observed diarrhea, or, conversely, if secretory diarrhea triggered the hypomagnesemia. In our case, the treatment was rapid correction of magnesium and a prolonged low-dose magnesium supplementation. While SGLT2 inhibitors have emerged as a potential avenue for managing hypomagnesemia, further research and clinical experience are necessary before they can be considered standard treatment. It needs to be kept in mind for clinicians that patients with certain carcinomas have to be monitored for electrolyte parameters as these may have PNS and may present with conditions such as hypomagnesemia.
